# Gd^3+^-Asparagine-Anionic Linear Globular Dendrimer Second-Generation G2 Complexes: Novel Nanobiohybrid Theranostics

**DOI:** 10.1155/2017/3625729

**Published:** 2017-09-26

**Authors:** Nasim Hashempour Alamdari, Mahmood Alaei-Beirami, Seyed Ataollah Sadat Shandiz, Hadi Hejazinia, Rahimeh Rasouli, Mostafa Saffari, Seyed Esmaeil Sadat Ebrahimi, Artin Assadi, Mehdi Shafiee Ardestani

**Affiliations:** ^1^Department of Radiopharmacy and Medicinal Chemistry, Pharmaceutical Sciences Branch, Islamic Azad University, Tehran, Iran; ^2^Drug Applied Research Center and Students' Research Committee, Tabriz University of Medical Science, Tabriz, Iran; ^3^Department of Biology, Central Tehran Branch, Islamic Azad University, Tehran, Iran; ^4^Department of Radiopharmacy, Faculty of Pharmacy, Tehran University of Medical Sciences, Tehran 1417614411, Iran; ^5^Department of Medical Nanotechnology, School of Medicine, International Campus, Tehran University of Medical Sciences, Tehran, Iran; ^6^Department of Medicinal Chemistry, Faculty of Pharmacy, Tehran University of Medical Sciences, Tehran, Iran

## Abstract

Designing a unique theranostic biocompatible, biodegradable, and cost-effective agent which is easy to be synthesized as a biohybrid material was the aim of this study. In this matter, asparagine attached to anionic linear globular dendrimer G2 (as a biocompatible, biodegradable, and cost-effective agent which is negatively charged nanosized and water soluble polymer that outweighs other traditionally used dendrimers) and finally contrast agent (Gd^3+^) was loaded (which made complexes) in synthesized asparagine-dendrimer. Observations revealed that, in addition to successful colon cancer and brain targeting, Gd^3+^-dendrimer-asparagine, the proposed theranostic agent, could increase T1 MR relaxation times, decrease T2 MR relaxation times significantly, and improve contrast of image as well as illustrating good cellular uptake based on florescent microscopy/flow cytometry and ICP-mass data. In addition to that, it increased tumor growth inhibition percentage (TGI%) significantly compared to FDA approved contrast agent, Magnevist. Totally, Gd3+-anionic linear globular dendrimer G2-asparagine could be introduced to the cancer imaging/therapy (theranostics) protocols after in vivo MR and fluorescent analysis and passing clinical trials. Hence, this nanotheranostic agent would be a promising candidate for brain drug delivery and imaging in the future.

## 1. Introduction

Cancer is one of the major causes of human mortality worldwide. According to WHO reports, breast/colon cancer is the most common cancer among humankind specifically women [1]. Improving sensitivity and specificity of the cancer detection and therapeutic methods in the primary stages could significantly aid in the patient's survival [2]. Nowadays, various methods are used to diagnose and treat cancer, such as imaging, biopsy, mammography, Pap smear, colonoscopy, blood tests, and novel chemotherapeutic agents based on biological biomarkers like amino acids. Molecular imaging is a valuable method in diagnosing and tracking of the treating stage and is recently known as a unique technique for monitoring of concurrent therapy and diagnostic agents called theranostics [3]. Generally compared, among various molecular imaging methods, Magnetic Resonance Imaging (MRI) could create higher contrast of the soft tissue versus Computed Tomography (CT) and is more economical versus Positron Emission Tomography (PET) [4–6]. MRI work is based on the presentation of hydrogen atoms in any tissues containing water molecules. MRI performance was evaluated by *T*1 (longitudinal relaxation time of surrounding water protons) and *T*2 (transversal relaxation time of surrounding water protons). In many procedures, MRI could not provide suitable contrast for diagnostic and staging aims [7, 8]. Gadolinium(III) is the most used contrast agent and works as a paramagnetic contrast agent (CA) to enhance the signal intensity in *T*_1_-weighted MRI. Performance of this parameter is determined by the relativity (*r*_1_) that is correlated to load capacity and the ability to freely contact with water. There are some reports reporting administration of Gd^3+^ under certain circumstances [9] and toxicity of gadolinium salts for mammalian addresses to make it chelate [10, 11]. Many studies proved that, among various chelating agents (as DTPA, DTPA-BMA, and HP-DO3A), superiority of nonionic and ring-shaped kinds with lower density and osmolality, by increasing the sensitivity of these CAs, is an important issue and requires much more effort [12, 13]. Otherwise, there is not any targeted contrast agent in medical market. These affairs address the use of dendrimers as a chelating agent.

Dendrimers are routinely synthesized divergent or convergent methods. By controlling the synthesis conditions, there is a possibility of achieving a dendrimer with desirable characteristics such as size (nm), shape, molecular weight, and even sometimes symmetric structures [14]. As indicated, dendrimers were classified as a vast class of nanosized polymers which have different charges on their surfaces like PAMAM as positive charge or anionic linear globular dendrimer (ALGD) as negative charge which have shown many medical advantages such as good water solubility in contrast to other polymeric nanoparticles like chitosan or PLGA and/or biodegrade/biocompatible/lower polydispersity properties which make them novel powerful drug delivery systems [4–8, 15]. Dendrimers have been showing promising applications in nanomedicine fields (drug delivery and imaging); dendrimer structure consists of three parts: (1) initial core; (2) interior layer (this part consists of repeated units linked to initial core. Radically, this layer shows the generation of dendrimer); (3) exterior layer (this layer is outer part of interior layer). Step by step, procedure and ability of dendrimer synthesis to choose different branching units provide exact control on polarity, monodispersity, shape, and generally physicochemical property [10, 15], symmetry, and spherical increase in higher generated dendrimer. This spherical structure with empty space and also high density of functional group (as amine and carboxyl group) on the surface of dendrimer promotes hydrophobic drug solubility, controls drug release, and makes dendrimer potentially suitable for targeted theranostic purposes. Most dendrimers have low toxicity compared to other nanoparticles. In addition, recent studies show that anionic dendrimers like ALGD have lower toxicity than cationic ones like commercially available PAMAM [16, 17]. Unique properties of tumors such as being hypervasculature, having defective vascular architecture, and having deficient lymphatic drainage system enhance the permeation and the retention (EPR) results in macromolecules specifically dendrimers accumulation and retaining in the tumor tissues [18]. It has been stated that dendrimer increases the cytotoxicity and the systematic half-life of chemotherapy drugs as well as imaging goals. In a study, gadopentetate was synthesized and attached to a negatively charged kind of dendrimer (consisting of PEG core and citric acid branches). Result showed that new synthesized CA was able to enter cells and increase the tissue relativity time and also had lower toxicity and higher efficiency; other similar reports also demonstrated such ability for positively charged dendrimers [6–10]. The main constraints that impede the application of dendrimers, such as PAMAM and PPI, include high toxicity, the difficulties in the synthesis pathway, and high production cost [17]. ALGD is negatively charged and highly available with facile and low price synthesis as well as fewer toxicity properties which recently developed and were biologically assessed successfully [15–18]. A very unique property of ALGD is its acceptable molecular weight (lower than 2000 Da) compared to other dendrimeric or nanosized polymeric structures which extensively prevent their structure from having immunological and toxicological features.

There are two important strategies in the design of ALGD; the first is the employment of polyethylene glycol (PEG) as a core interesting point of PEG is being biodegradable and having a low price and the ability to accumulate in tumor tissue without targeting agent [15–18]. Secondly, surface modification, for instance, with acetic acid, can be considered as a convenient method of synthesis. The studies on the synthesis of pegylated dendrimer, due to the unique characteristics such as having good availability, being biodegradable, and being inexpensive, were selected. In addition, due to the citric acid molecule being completely metabolized in human body, citric acid was chosen as surrounding group (to produce an anionic linear globular dendrimer, ALGD). So there is no concern about the safety of the nanoparticle in body. In other words, acetic acid groups, with negative charge, are located surrounding the PEG; the negative charge of dendrimer prevents cell membranes damage. Since such kind of negatively charged dendrimer is comprised of PEG and citric acid, the results may be presented as a novel biodegradable and biocompatible nanovector in all theranostic strategies [19–21].

In general, nanosized dendrimers are the novel generation of carriers able to supply water solubility, to reduce toxicity, and consequently to improve diffusion rate and bioavailability and thermodynamic and kinetic stability for any theranostics purposes. They can be targeted easily. Special ALGD characteristics like ability to attach to many molecules and having higher potential to find target make them a new attracting field for researchers. In corporation with Gd^3+^ to be of nanoscale, structures can enhance *r*_1_. Nanoparticulate shape, lower *r*_2_/*r*_1_ value, and monodispersibility in water may cause a higher signal-to-noise ratio and better anatomic resolution in T_1_-weighted images. In this respect, porous nanocarriers improve water accessibility to intraparticular Gd^3+^ [22]. Nanodendrimers are not targeted by themselves. It has been reported that targeting could decrease consumed drugs and side effects and increase efficacy [23]. Expression of many biochemical molecules such as silicic acid, folic acid, and receptors-N-methyl D-aspartate (NMDA) increases in tumor cell membrane and is a potential molecular target for tumor-selective drug delivery [24]. Body neoplastic changes, accompanied with high ratio of protein synthesis and result, increase the requirement of essential and nonessential amino acids in malignant growing cells [25, 26]. Otherwise, most of the NMDA receptors exist in central nervous system but the study has shown the existence of NMDR in some sort of cancer such as breast, ovary, and prostate cancer [27]. Folate-conjugated PAMAM-TU-DTPA Gd(III) uptake into folate receptor positive cell line by receptor-mediated endocytosis doubly increased the longitudinal relaxation rate [28].

Theranostics is called a class of new biochemically designed materials able to produce both therapeutic and diagnostic purposes concurrently. In this field nanoparticles because of unique role in biological systems have the major part of research on theranostics [2–8]. Additionally it has been carefully shown that gadolinium ions can be able to induce a significant apoptotic intercellular response when they entered into the cells [11, 12a; 12b, 13-17] and this is a key point to introduce gd^3+^ ions as powerful antimalignant cells if they were delivered selectively. Two approaches, including increase in N-methyl-D-aspartate receptor expression in cancerous and brain cells for trapping nitrogen, selected as targeting choice and asparagine as ligand, and entrapping gadolinium ion into cancerous cells as intracellular apoptosis inducer, were selected to design the proposed dendrimeric structure of asparagine loaded gadolinium ions to make a good and effective theranostic agent. Based on the above finding, it has been hypothesized that nanosized targeted contrast agent could improve signal intensity and consequently improve cellular uptake as well as image resolution and concurrent antitumor activity and also decrease the side effects on normal human kidney cells as major target site of gadolinium contrast agents. Based on this hypothesis in this study, novel chemically designed theranostic Gd(III)-anionic linear globular dendrimer-asparagine was developed and assessed for MR imaging and therapy of metabolically active tissue. In brief, the current study showed a novel gadolinium based amino acid conjugated negatively charged dendrimer with no more significant toxicological features in vitro and in vivo; otherwise a very promising anticancer activity was observed in addition to successful tumor detection as well.

## 2. Experimental Section

### 2.1. Chemicals, Instrumental Analysis, Cells, and Medium

Polyethylene glycol, citric acid anhydrous, sodium bicarbonate, and methanol were purchased from Merck. Solvents and chemical reagents were of analytical grade from Merck. Gadolinium(III) chloride (99.99%), asparagine, and sephadex were obtained from Sigma-Aldrich. Chemical agents were used in the study.

FTIR (Nicolet), NMR (Bruker), zeta sizer (Nano ZS, Malvern), CHN analyzer (SERIES II, Perkin Elmer), liquid chromatography-mass spectroscopy (Bruker), Lyophilizator (Wisco), ELISA reader (Bio-Tek), and ICP-AES (Varian AX150 turbo) devices were used in the study. All the cells provided for the study including HEK-293 and HT-29 were purchased from National Cell Bank, Pasteur Institute of Iran. The culture medium was elected as DMEM that contains 5 ml FBS 10%, 1% of a mixture of penicillin and streptomycin, and 5 mL of glutamine provided from Sigma-Aldrich. After enough cells' growths, they were treated with trypsin enzymes and separated cells were centrifuged at 2000 rpm for 5 minutes.

*Animals*. All the subjected animals were provided by Pasteur Institute of Iran and kept normally and used in accordance with the ethics of animal guidelines provided by universal policies such as Declaration of Helsinki and ethical committee of Tehran University of Medical Sciences under the project number 94-01-159-28586 as well.

### 2.2. Nanocontrast Media Synthesis

Nanocontrast agent synthesis procedure consists of three main steps: (1) synthesis of the anionic linear globular G_2_ dendrimer (based on development of Namazi et al. with enough modifications regarding green chemistry approaches like replacing thionyl chloride with carbodiimide family and decreasing the reaction time and kind of purification), (2) conjugation of the asparagine amino acid to the ALGD, and (3) gadolinium loading on synthesized nanostructure molecule.

### 2.3. Anionic Linear Globular Dendrimer (G2) Synthesis

**Figure 1:**
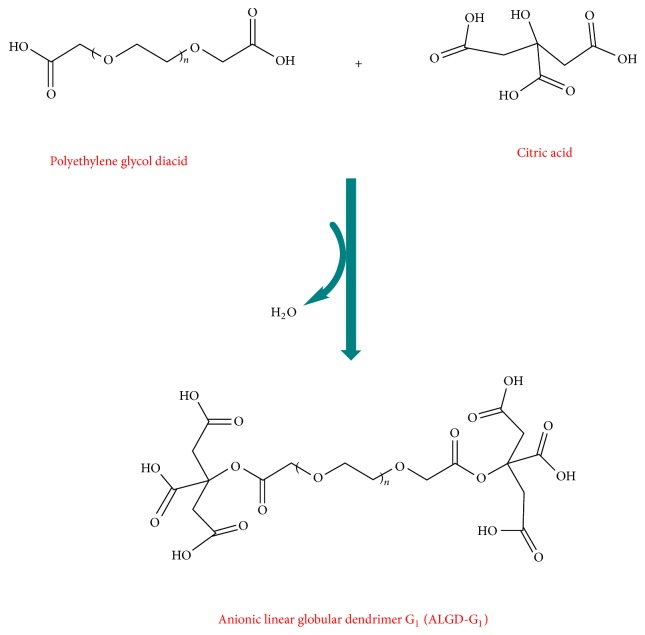
Dendrimer G1 synthesis.

**Figure 2:**
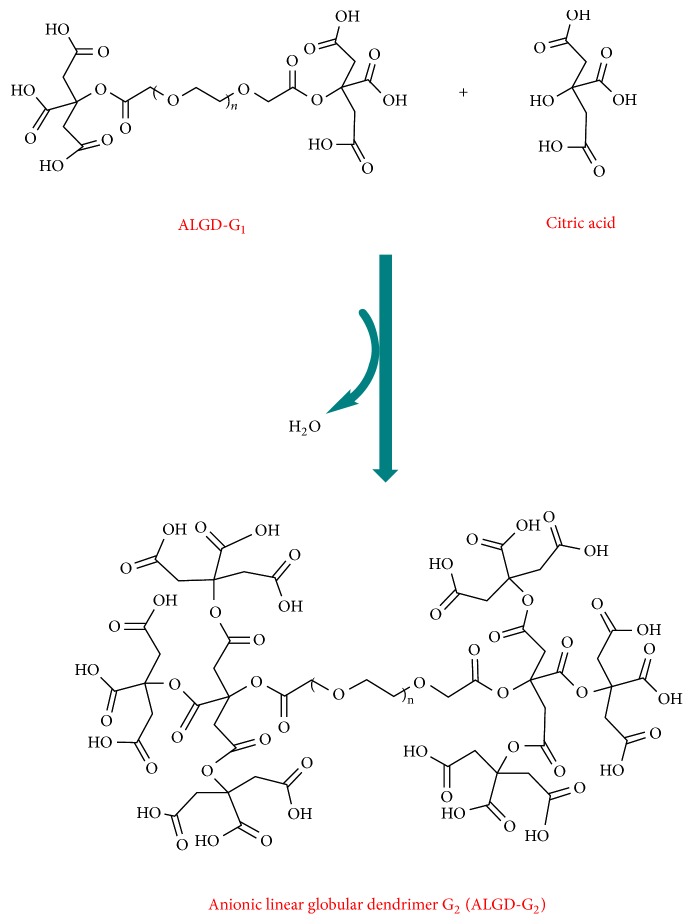
Anionic linear globular dendrimer G2.

Polyethylene glycol (PEG) with two carboxyl groups on the sides formed the core and citric acid and formed interior and exterior layers of the dendrimer. Engineered polyethylene glycol (5.65 mmole) was added to the dried dimethyl sulfoxide (DMSO). DMSO worked as a basis of the reaction. N,N′-Dicyclohexylcarbodiimide (DCC) (2 mmole) was added to activate the carboxyl functionality and was mixed for 51 minutes. Now terminated carboxyl groups of PEG are ready to interact with citric acid's carboxyl group to form ALGD G1. Then 5.2 mmole anhydrous citric acid was added to the reaction container and stirred on 400 rpm for 3 hrs. This step yields the first generation of the dendrimer. In the next step to synthesize the second generation, at first we activated terminated assembled citric acid. 3 mmole DCC was added to the latter and stirred for at least 51 minutes followed by an addition of 10.4 mmole. Anhydrous citric acid was stirred in room temperature for 72 hours in capped container when a dark brown color developed. The color change verified the synthesis of the G_2_ dendrimer (Figures 1 and 2).

### 2.4. Purification of ALGD G2

Presence of impurity could interact with the next step of synthesis so that chromatography against sephadex and lyophilization are used as a purification method. The compact sephadex G75 phase retained the DCC and citric acid. The resultant dendrimer was rechromatographed against sephadex to achieve high purity grade of the samples. Then the samples were lyophilized for DMSO removal.

### 2.5. Confidence of ALGD G2 Synthesis

HNMR, FTIR, and CHN analysis were carried out to confirm the structure and zeta sizer was used to determine the charge, size, and molecular weight.

### 2.6. Asparagine Conjugation

**Figure 3:**
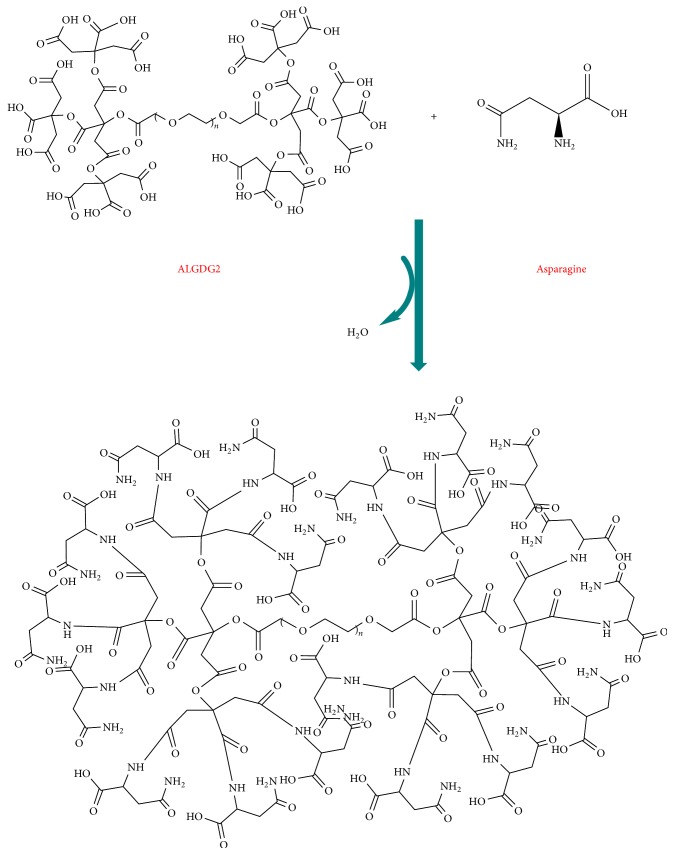
Anionic linear globular dendrimer G2-asparagine.

First the G_2_ lyophilized dendrimer and DCC or EDC were stirred for at least 15 minutes to activate functional groups of ALGD G2 to make it ready for conjugation with protected asparagine. Then protected asparagine with a molar ratio of 2 : 1 was added to activate dendrimer and was stirred on 300 rpm for 72 hours. Then, asparagine conjugated to ALGD2 (Figure 3).

### 2.7. Asparagine Purification

Chromatography against sephadex-G75 and lyophilization were used as the previous step to yield purity. The obtained conjugate was then deprotected and afterwards purified as described.

### 2.8. Confidence of Asparagine Conjugation

TLC, HNMR, and FTIR analysis were carried out to confirm the structure.

### 2.9. Gadolinium Loading on the Dendrimer

**Figure 4:**
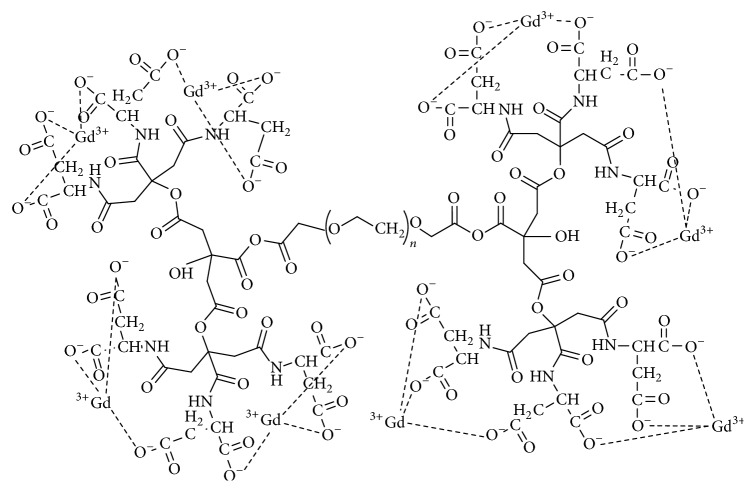
Gd^3+^-anionic linear globular dendrimer G2-asparagine.

Asparagine-dendrimer conjugate (2.01 mg) was dissolved in distilled water and sodium bicarbonate was added to make the sample alkaline and consequently to increase mixture ionization followed by 15 minutes of stirring. Gadolinium chloride (20 mg) was added to the mixture and was sealed. The sample was on heater stirrer for 10 minutes in 100°C and then stirred on 300 rpm for 6 days with light exposure protection for gadolinium loading (Figure 4).

### 2.10. Confidence of Gd^3+^ on ALGD G2-Asparagine

Free gadolinium is severely poisonous and consequently this purification step mainly affects synthesized nanocontrast media safety; so the sample was then purified by column chromatography against sephadex and dialysis (500 Da cut-off).

### 2.11. Measurement of Loaded Gadolinium

Gadolinium loading was assessed using ICP-mass spectroscopy.

### 2.12. Real-Time PCR with SYBR Green

A SYBR Green real-time quantitative PCR was performed to assess the expression of* Bax* and* Bcl-2* gene in HT29 cell line. Total cellular RNA was extracted from the treated and untreated cells using the RN easy Plus Mini Kit (Qiagen) according to the manufacturer's protocol. High quality of RNA was isolated for cDNA synthesis by using Quanti-Tect Reverse Transcription Kit (Qiagen) according to the manufactures' instructions. Primer specificity for real-time PCR was tested utilizing BLAST program (https://blast.ncbi.nlm.nih.gov/blast). Each real-time PCR reaction was performed in a total volume of 20 *μ*l reaction mixture according to the instruction of DNA master SYBR Green mix (Roche Applied Sciences). The primer concentrations were adjusted to 0.4 *μ*M for genes. The cycling parameters were 10 min at 95°C, 5 mins at 95°C for denaturation, and 15 s at 95°C, followed by 60°C for 1 min in PCR cycling condition. Amplification stage was followed by a melting stage: at 95°C for 20 s, 60°C for 60 s, and 95°C for 20 s in an ABI 7300 real-time PCR system (Applied Biosystems, USA). The gene expression was calculated using comparative threshold cycle (Ct). Subsequently, mean threshold cycle (mCt) value of internal housekeeping gene (*GAPDH*) was subtracted from mCt value of the target genes to achieve ΔCt and ΔΔCt. Values of each sample were measured from corresponding Ct values; the mRNA level acquired for each sample was normalized to that of human glyceraldehydes-3-phosphate dehydrogenase (GAPDH) mRNA level. Eventually, target/internal control gene expression ratio was measured according to the ratio formula (Ratio = 2^−ΔΔCt^).

### 2.13. In Vitro MRI Imaging

MRI imaging was performed by using a 1.5 Tesla General Electric (US) piece of device in the Rajaei Cardiac Center Hospital, Tehran, Iran.

Gd^3+^-ALGD G_2_-asparagine conjugate was prepared in various concentrations (0.22, 0.11, 0.055, 0.0275, 0.0137, and 0.00687 mg/ml). Distilled water as the blank was used and magnetizing was used as the standard sample with equal doses. *T*1 and *T*2 weighted MRI at 1.5 T intensity were performed on phantom axial slices, and according to resultant imaging suitable concentration was estimated.

### 2.14. Imaging Protocol

Multipoint method was used to obtain *T*_2_ through analysis of exponential curve of signal intensity against echoes of four spin eco sequences based on TE. The protocol is illustrated as follows.

Standard spin echo: number of echoes = 4, TE = 22/44/66/88/110/132/154/176/198/220/242/264/286/308/330/352 ms, TR = 3000 ms, matrix = 512*∗*384, slice thickness = 4 mm, FOV = 25 cm, NEX = 3, and pixel band width = 130.

Signal intensity in a four-spin echo protocol is as follows:

(1) SignalSE4TE,T2=S0e−TE/T2⟹ln⁡SignalSE4=ln⁡S0−TET2.

Therefore regression of the linear curve on the natural logarithm of signal intensity against TE yielded slope of −1/*T*2 by excel. *T*_1_ was obtained using multipoint method and analysis of exponential curve of signal intensity against TR; the following protocol was used.

Standard spin echo: number of echoes = 1, TE = 15 ms, TR = 32/50/100/200/400/600/1000/2000/3000, matrix = 512*∗*384, slice thickness = 4 mm, FOV = 25 cm, NEX = 3, pixel band width = 130.

### 2.15. Intracellular Uptake Study

Intracellular uptake of Gd3+-anionic globular dendrimer G2-asparagine was determined by the following method: In the first step, concentration of 2 × 10^5^ cells per well was replaced and incubated at 37°C and 5% CO2 for 24 hours. In three wells, considered as control, 200 *λ* Magnevist drug (as a positive control) and 200 *λ* Gd3+-anionic globular dendrimer G2-asparagine were added to them. After incubation of cells at 37°C with 5% CO2 for 60 min, washing them was done twice with 500 *μ*L of phosphate buffer saline and then they were centrifuged at 1500 rpm for 15 minutes and reconstitution was done in 2 mL of cell culture media.

Quantitative determination cellular uptake of Gd3+-anionic globular dendrimer G2-asparagine was done by ICP-AES. These measurements were performed in triplicate and the mean and standard deviation of the results were calculated.

For more confirmation Fluorescent Cellular Uptake Assay was also performed in which the experiment was carried out by loading BODYPI fluorescent dye in Gd3+-anionic globular dendrimer G2-asparagine and then HT29 cells were exposed for 60 min. Finally cellular imaging was taken by normal and fluorescent microscopes.

### 2.16. Blood Clearance Half-Life

A calculated amount of Gd^3+^ ions of nanocontrast (0.5 mmole Gd^3+^-asparagine-dendrimer) was injected into veins of mice (male, *n* = 6) and at different times after injection blood samples of animals were collected and Gd^3+^ ion was elaborately determined by ICP-AES up to 140 min after injection to calculate half-life of blood clearance of such nanoconjugate.

### 2.17. In Vitro Toxicity Assay

A widely used method for cell viability measurement is MTT assay; viable cells reduce MTT to formazan. In the first step, the cells (HT29) were incubated with various concentrations of the conjugates for 24 hrs. Then the supernatants of the cells were removed. The final concentration of 0.5 mg/mL MTT solution was added to each well of the plate and after the cells' incubation for an additional 4 hours.

The solutions were removed, and the dye was dissolved in 100 *µ*l dimethyl sulfoxide with glycine-NaCl buffer; the plate was placed in a dark place for one hour in order to be ready for spectrophotometric determination. The amount of absorption in each well was calculated by an automated microplate reader at 570 nm. The results showed the absorption of the cells and are expressed as percentage of viable cells.

### 2.18. In Vivo Imaging

Two male Syrian mice weighing about 18 g (8 weeks old) were used for in vivo imaging as breast cancer animal model (MCF-7 xenograft model). The mice were anesthetized by 0.1 ml of ketamine and xylazine mixture prior to the procedure. The mice were anesthetized completely after 3 minutes and then were fixed on the imaging film using tape. Then total body imaging was performed using 1.5 T MRI devices. Then 0.2 ml/kg nanosized sample was injected intravenously. After the injection, imaging was repeated on total body to compare the resolution and contrast of the images.

### 2.19. In Vivo Efficacies

Explaining in vivo growth effectuality with chemically designed nanoconjugate, the experiment was administered on murine colon tumors CT26 which noninheritable from the Louis Pasteur Institute of Persia. Cells were refined and preserved at 37°C in an exceedingly humidified setup with five-hitter greenhouse emission. The cell medium is composed of Dulbecco's changed Eagle's medium (DMEM), 100% craniate bovine humour, a pair of millimetre L-glutamine, a hundred U/mL antibiotic drug, and a hundred *μ*g/mL antibiotic. Nine feminine BALB/c mice (20 ± a pair of g, 6–8 weeks old) were noninheritable from the Louis Pasteur Institute of Persia and chosen for the growth model. During this work, BALB/c mice were divided into 3 teams and unbroken in an exceeding status with the twelve h light/dark cycle at 23 ± 2°C temperature. Animal study was conducted in line with national rules and approved by the Animal Experiments Moral Committee. Throughout growth implantation, mice were anesthetized with isoflurane (1.4% atm); CT26 growth cells were suspended at a density of two × 10^5^ cells/mL in DMEM cell medium. To establish a colon cancer CT26 model, the cells suspension (50 *μ*L/mouse) was subcutaneously inoculated to inject within the back of 3 teams of feminine sport BALB/c mice. Estimating growth size (TS) was performed with a caliper (length × dimension × height). One week is concerned till solid tumors reach a spread from fifty to a hundred mm^3^. Receiving nonidentical remedy, all tumor-bearing mice were randomly divided into several groups and were injected via tail vein one time each. The injection was performed with saline (control), Magnevist (200 *µ*g Magnevist per metric weight unit of mouse weight), and nanoconjugate (200 *µ*g per metric weight unit of mouse weight). Growth sizes and mouse body weights were calculated every five days. Growth volumes were normalized to 100% at day zero and exploitation was calculated using the subsequent formula: *V* (mm^3^) = 1/2 length (mm) × width (mm^2^) [11–13].

On day 10, all animals were euthanized. Tumors were excised to calculate tumor growth inhibition (TGI), which was measured as TGI = (1 − (mean tumor weight of treatment group)/(mean tumor weight of control group)) × 100% [12, 13].

### 2.20. Histopathological Assessment

Days days after imaging dosage injection of both fluorescent and nonfluorescent nanoconjugated Gd3+-anionic globular dendrimer G2-asparagine, each receiving animal was ethically executed and kidney and liver as vital organs were removed and fixed by formalin and histopathological lams were prepared by staining with H&E to investigate any significant side effects.

### 2.21. Statistical Analysis

Statistical data analysis and *T*_1_ and *T*_2_ measurements were done using Dicom software, Matlab, and excel software. Micro-Dicom software package was also used for image observation. SPSS was used for quantitative data analysis while paired *t*-test with Turkey post hoc and one-way ANOVA, in case of cluster comparison, were applied.

## 3. Results and Discussion

### 3.1. Nanocontrast Media Synthesis

Engineered PEG core carrying two terminated carboxylic acid groups reacted with citric acid molecules and led to G1 dendrimer synthesis by forming diketo ester bonds. Then, hydroxyl groups of citric acid molecules react to carboxyl moieties of the G_1_ dendrimers and yield the G_2_ dendrimer.

The free amine functional group of the asparagine reacted to carboxyl groups of the G_2_ dendrimers and then yielded G_2_ dendrimer-asparagine molecule by forming amide bonds.

In the final step, the gadolinium was loaded on the synthesized G2 dendrimer-asparagine structure.

### 3.2. TLC (Thin Layer Chromatography)

TLC is a simple, quick, and inexpensive procedure that gives the chemist a quick answer as to how many components are in a mixture. TLC is also used to support the identity of a compound in a mixture when *R*_*f*_ (retention factor) of a compound is compared to *R*_*f*_ of a known compound (preferably both run on the same TLC plate).

**Figure 5:**
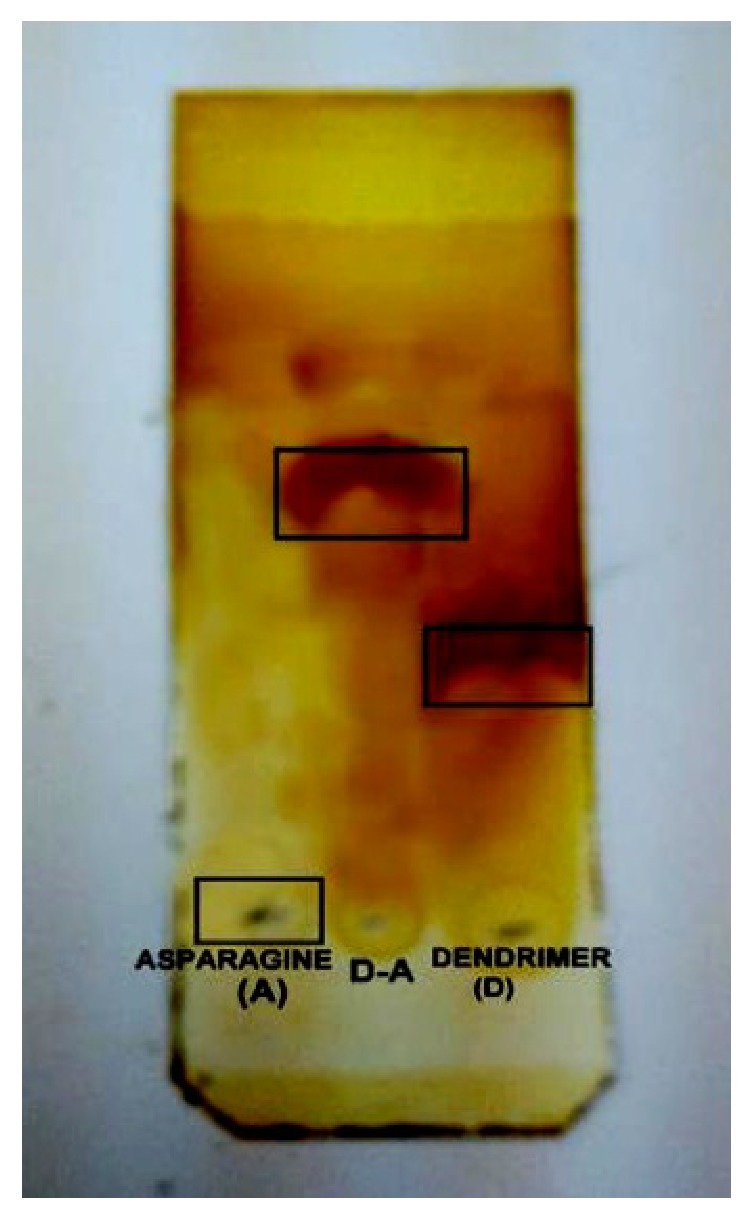
The spots on TLC paper after exposure to iodine.

TLC profiling has been done to confirm satisfactory reaction between G_2_ dendrimer and asparagine. (Table 1, Figure 5).

RF parameter's calculation results verified that *R*_*f*_ for dendrimer, asparagine, and dendrimer-asparagine emphasizes dendrimer-asparagine complex's establishment.

### 3.3. Gadolinium Content Determination

Determination of gadolinium content showed that the gadolinium ion was loaded on the G_2_ dendrimer-asparagine structure at a concentration of 220 mg/L (Table 2).

According to 20 mg Gd^3+^ being consumed in 10 ml of dendrimer-asparagine solution, finally 22 mg of Gd^3+^ was determined in 1000 ml of solution; this report showed loaded gadolinium is equal to 11 percent.

### 3.4. Spectrometry Results

UV, FTIR, HNMR, and LC-mass spectrometry have been done to emphasize dendrimer and dendrimer-asparagine synthesis.

### 3.5. UV Spectrometry

**Figure 6:**
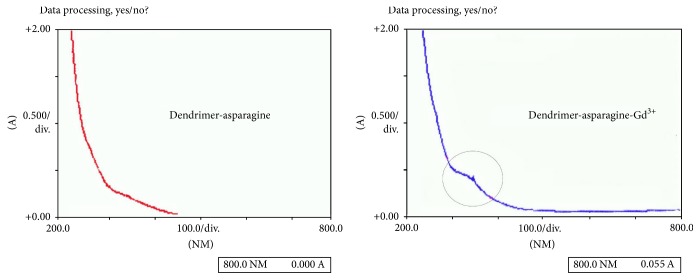
The UV spectrum of dendrimer-dendrimer-asparagine.

Although UV spectrometry could not be used to confirm synthesis and identification of unknown compound lonely, we used UV spectrometry as auxiliary method to confirm asparagine conjugation as can be seen in Figure 6.

UV spectroscopy showed an absorbance at about 150 nm in the Gd^3+^-G_2_ dendrimer-asparagine that was absent in the G_2_ dendrimer-asparagine so that the gadolinium loading and complex formation were denoted.

### 3.6. FTIR Spectroscopy

**Figure 7:**
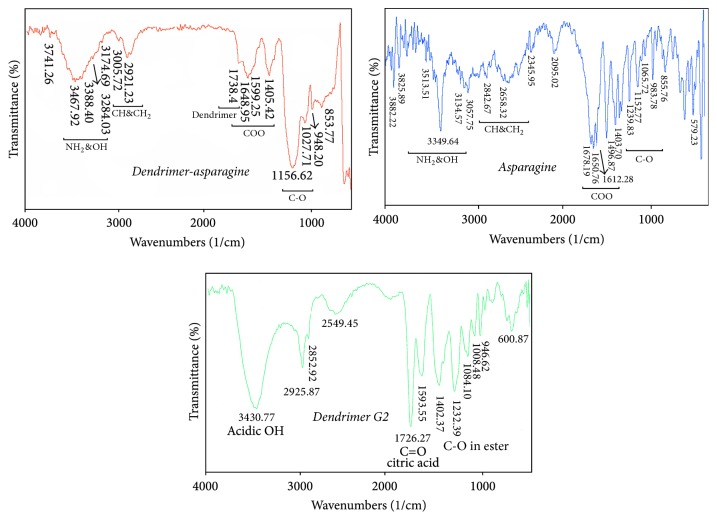
Dendrimer-asparagine and asparagine as well as dendrimer G2 FTIR spectrums, respectively.

Like fingerprint, no two unique molecular structures produce the same FTIR spectrum. This makes FTIR spectroscopy useful for several types of analysis. Resulting spectrum of dendrimer G2 and asparagine is compared with dendrimer G2-asparagine spectrum and is shown in Figure 7.

The peaks between 3200 and 3600 cm^−1^ are related to NH and OH groups. The peak 1590–1760 wavelength shows carbonyl groups of compound, aliphatic carbon-hydrogen peaks at 2600–3000 cm^−1^ that are common between the asparagine and asparagine-dendrimer, and 1738.4 cm^−1^ peak that exclusively depicts the dendrimer. This also exists in dendrimer G2-asparagine FTIR spectrum (Figure 7).

### 3.7. ^1^H-NMR Spectroscopy

**Figure 8:**
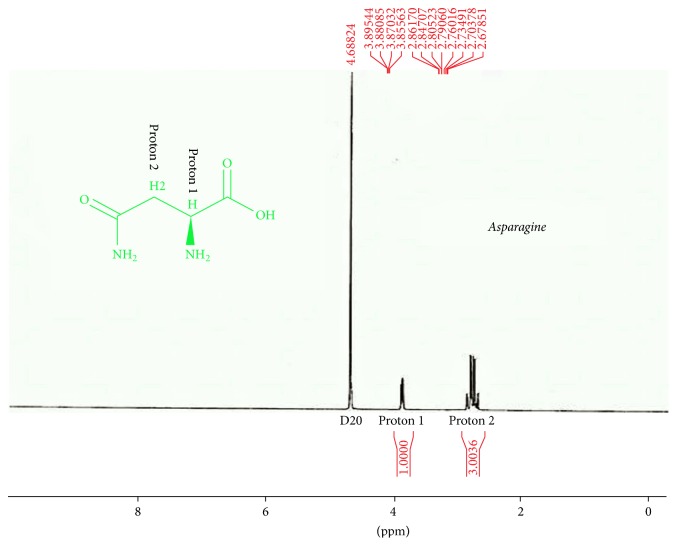
Asparagine HNMR outlook view.

**Figure 9:**
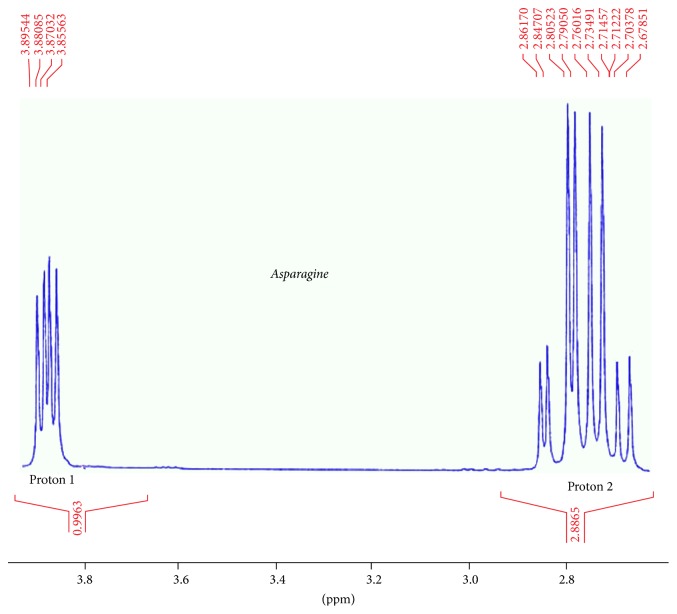
HNMR of asparagine.

**Figure 10:**
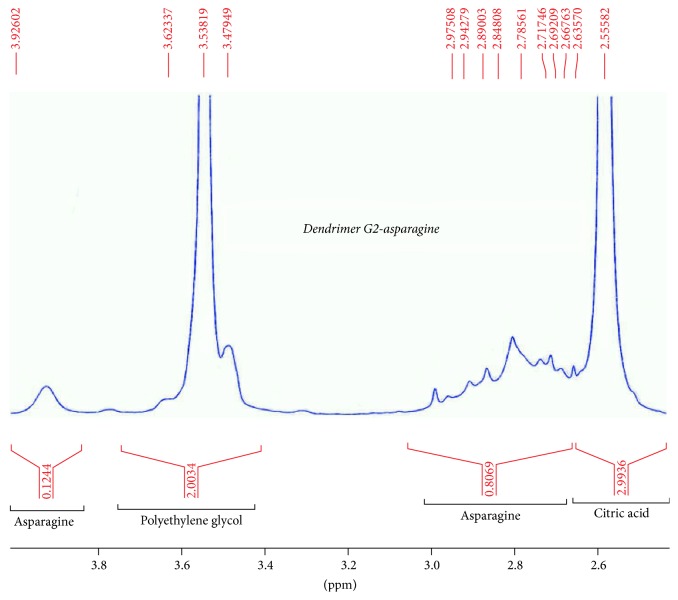
Dendrimer-asparagine HNMR result.

^1^H-NMR spectroscopy was performed using D_2_O solvent. Therefore, OH and NH_2_ peaks were not detectable. Asparagine HNMR spectrum was used for better interpretation of dendrimer G2-asparagine HNMR spectrum. The chemical shift in 4.6882 is related to D2O solvent. Two main peaks are presented in asparagine HNMR spectrum; the chemical shifts in (2.8–2.9)*δ* are related to proton 1 which is adjacent to carboxylic acid. Electron attraction effect of carboxylic acid causes ^1^H absorption in lower electrical field and higher chemical shift in comparison to ^2^H adjacent to amide group. The chemical shifts in the asparagine-dendrimer spectrum contain the 2.8 and 3.9 ppm peaks that are identical to those of the asparagine structure. The chemical shifts *δ* 2.5 and 3.5 *δ* are related to the citric acid and polyethylene glycol moieties in the dendrimer structure, respectively. These chemical shifts verify the synthesis of the asparagine-dendrimer molecule. The results are depicted in Figures 8–10.

### 3.8. LC-Mass Spectroscopy

**Figure 11:**
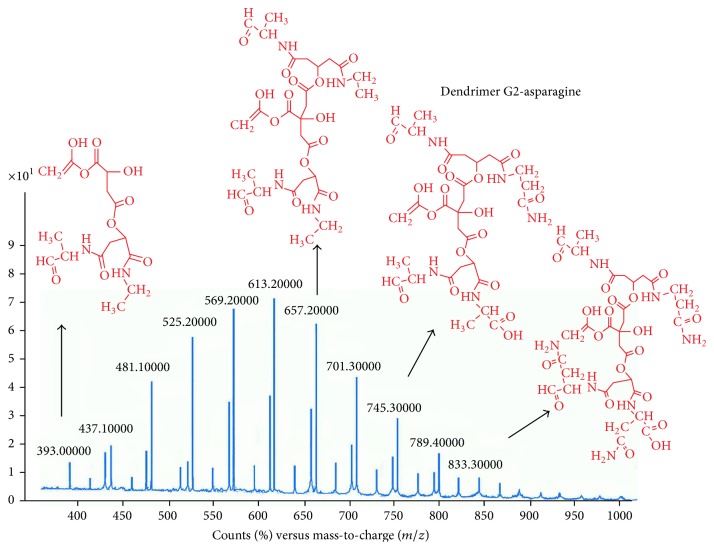
LC-mass result on negative and positive mode.

LC-mass spectroscopy was used in synthesized nanostructure carried out in positive and negative mode (Figure 11).

Its application is oriented toward the separation and general detection of particular mass in substance. The molecular weight of the original Gd^3+^-dendrimer G_2_-asparagine molecule is 3096 Da that was evident through the molecular fragments observed. However, many fragments were justified regarding the McLafferty fragmentation mechanism for the amide and ester functionalities.

### 3.9. CHN Analysis

CHN analysis result indicates approximate atomic percentage of synthesized nanocontrast agent. The results were justified with respect to PEG core poly-dispersive characteristics and variable linking of citric acid branches.

Result of dendrimer G2-asparagine CHN analysis is shown in Table 3 and compared to dendrimer G2 alone. The data showed an increase in carbon and hydrogen and nitrogen contents of nanoconjugate and led to confirming a new derivative from dendrimer structure.

### 3.10. Size and Charge and AFM Imaging

**Figure 12:**
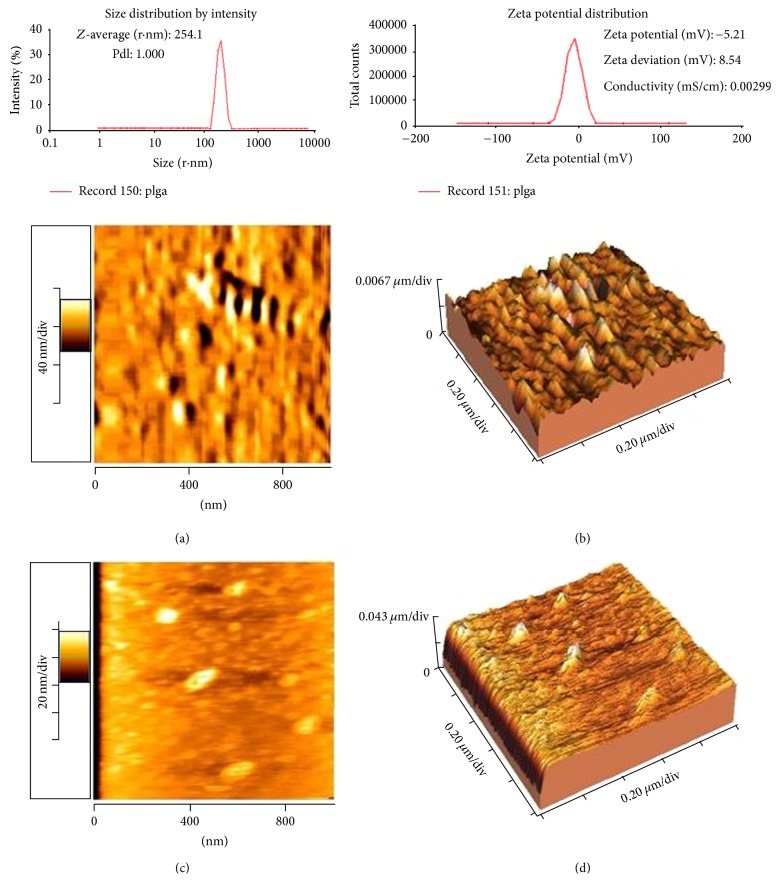
Size and charge measurement for Gd^3+^-dendrimer G2-asparagine. It is well previously [20, 21] shown that dendrimer alone has -1-2 mV charge and ~90 nm size. The data was also confirmed by AFM imaging results as depicted above: (a) Gd^3+^-asparagine-dendrimer G2, 2D image; (b) Gd^3+^-asparagine-dendrimer G2, 3D image; (c) dendrimer G2, 2D image; (d) dendrimer G2, 3D image.

Using zeta sizer instrumentation showed that the molecular size and charge have experienced significant change following the asparagine conjugation. Data showed a significant increase in both size and zeta positional of nanosized conjugate compared to anionic linear globular dendrimer G2 molecule. The results were also confirmed by AFM imaging outcomes (see Figure 12).

### 3.11. Cellular Uptake

**Figure 13:**
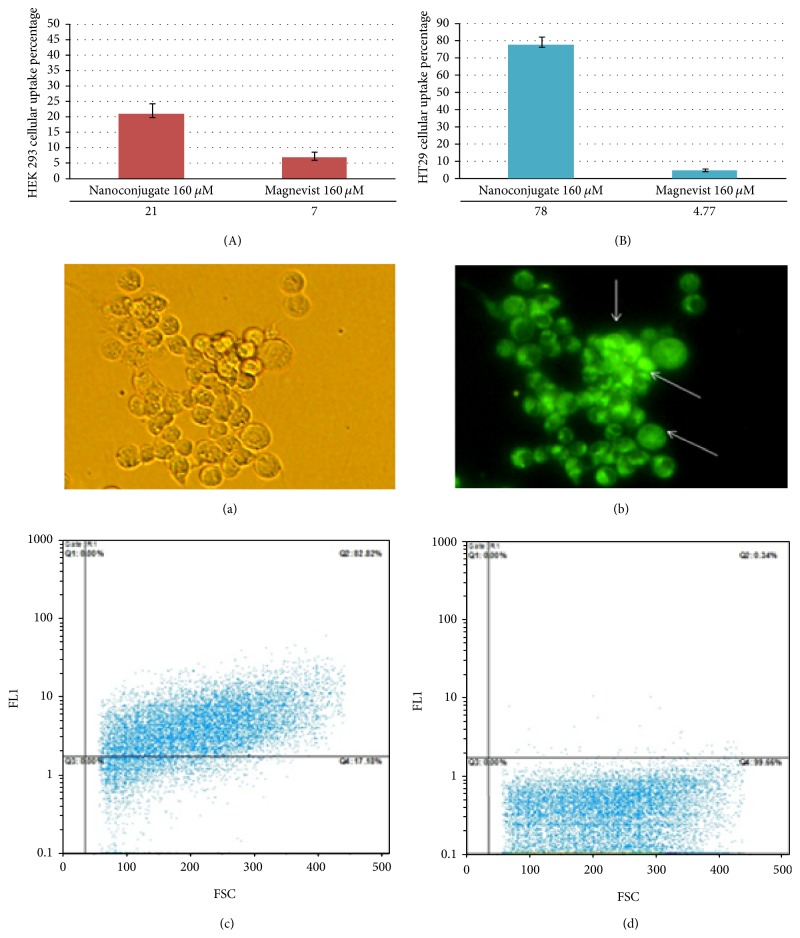
Data obtained regarding the percentage of gadolinium ion cellular uptake in (A) normal human kidney cells (HEK293) and (B) HT29 cancerous cells as well. Flow cytometry and microscopic imaging 60 min after exposure (a), (b) Gd^3+^-asparagine-dendrimer G2 as qualitative image, (c) Gd^3+^-asparagine-dendrimer G2-89% cellular uptake, and (d) was considered as being immediately possible after exposure (<7%) as depicted below.

Magnevist is not able to penetrate a cell, so it needs to use an appropriate carrier. One way, by attaching the magnevist to dendrimer, makes it possible to enter intracellular space. As Figure 13 appropriately shows that the amount of the synthetic contrast agent could enter the HT29 cell line. Penetration ability of this new synthetic contrast agent is probably due to its nanosize. Receptor-mediated endocytosis is the penetration mechanism.

As the above data show, it is concluded that Gd^3+^-dendrimer G2-asparagine demonstrated a very good and significant *p* < 0.01 cellular uptake in both normal and cancerous cells but with different ratios compared to Magnevist (results obtained after 6 hrs exposure).

This experiment was carried out by loading BODYPI fluorescent dye for 60 min after exposure (a), (b) (Gd^3+^-asparagine-dendrimer G2) as qualitative image, (c) (Gd^3+^-asparagine-dendrimer G2-89% cellular uptake), and (d) was considered as being immediately possible after exposure (<7%). This experiment was performed on HT29 cells by flow cytometry apparatus as well as microscopic imaging (results obtained after 6 hrs exposure) (see full details in Figure 13).

### 3.12. MTT Assay

**Figure 14:**
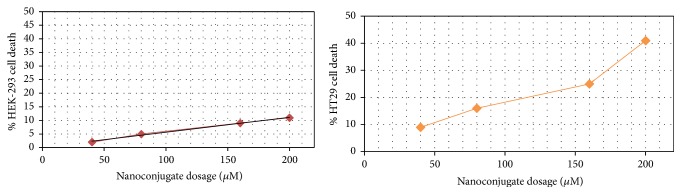
Results of Gd^3+^-dendrimer G2-asparagine MTT assay on HEK-293 normal kidney cells and HT29 cancer cells. The results demonstrated a significant toxicity for nanoconjugate on HT29 cancer cell line whereas nonsignificant toxicity was observed on HEK293 cells versus control group (100% cell viable data not shown).

**Figure 15:**
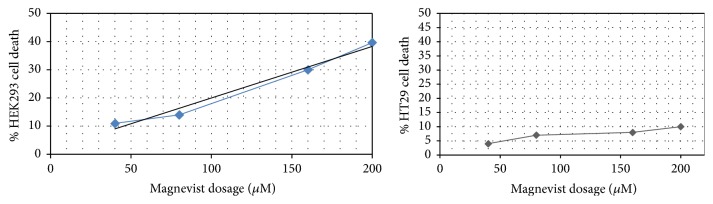
Results of Magnevist MTT assay on HEK-293 normal kidney cells and HT29 cancer cells. The results demonstrated a significant toxicity for Magnevist on HEK293 human normal kidney cell line whereas nonsignificant toxicity was observed on HT29 cancer cells versus control group (100% cell viable data not shown).

The Gd^3+^-dendrimer G2-asparagine conjugate was seen more toxic than the Magnevist in all cell lines after 24 hrs exposure (as can be seen in Figures 14 and 15).

A total comparison between the results indicates that cytotoxicity (in HT29 cell line) is increased due to exposure of nanosized conjugate and decreased toxicity was observed in normal cells exposed to nanosized conjugate compared to control drug Magnevist which was found more toxic than nanoconjugate on normal human kidney cells.

Based on cellular toxicity data it is concluded that Gd^3+^-asparagine-anionic linear globular dendrimer-G2 is a very promising good theranostic radio-opaque agent comprising nanosized pegylated asparagine as tumor attracting molecule. Compared to Magnevist the nanosized molecule showed safe effects on human kidney cells as major targets of gadolinium based contrast agents.

### 3.13. Relaxation Time Evaluations (In Vitro MRI Imaging)

Table 4 shows the signals obtained from different concentrations and TR times. T1 was determined by using the linear equation and compared with Magnevist and water. T1 (SP-1) shows the relaxation recovery time and different drug concentration effects on the T1 relaxation time prolongation.

The studies revealed simultaneous increase in T1 and decrease in T2 time periods while the highest signal intensity alterations were observed in 0.055 and 0.0275 concentrations.

### 3.14. Gene Expression Assay

**Figure 16:**
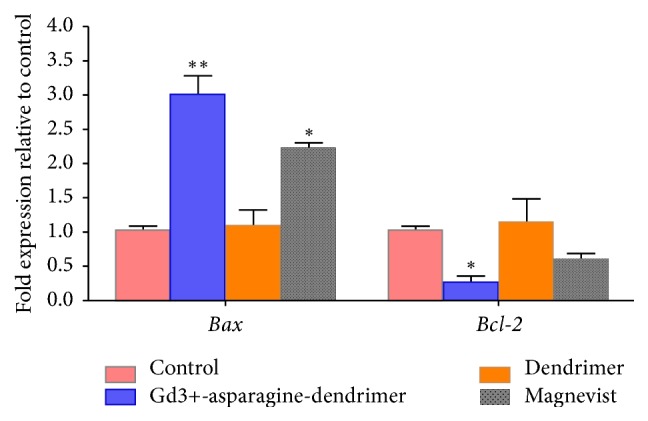
Impact of dendrimer, Magnevist, and Gd3+-dendrimer-asparagine conjugate at a concentration of 160 *µ*M in expression of Bax and Bcl-2 mRNA levels in HT29 cells. The expression of mRNAs was analyzed by real-time PCR and normalized by* GAPDH* expression. *P* value less than 0.05 was considered statistically significant for one-way ANOVA analysis followed by Student's *t*-test. ^*∗*^*P* < 0.05, ^*∗∗*^*P* < 0.01 compared to control.

To verify the expression of apoptotic and antiapoptotic genes at mRNA level in the samples exposed in HT29 cell line using quantitative real-time PCR (Figure 16), our data demonstrated that mRNA level of Bax was significantly upregulated while the expression of antiapoptotic Bcl-2 was significantly downregulated in cells treated with Magnevist and dendrimer conjugate compared to untreated control. These effects could be attributed to an increased sensitivity of HT29 cell line to induced apoptosis when exposed to Gd3+-asparagine-dendrimer conjugate. Therefore, the Bax/Bcl-2 ratio increased.

### 3.15. Blood Clearance Half-Life

According to the obtained data half-life of Gd^3+^ ion clearance average was found at 76.13 min based on the obtained equilibrium (the data was shown at SP-2). Nanocontrast agent showed a rapid clearance from the blood.

### 3.16. In Vivo MR Imaging

**Figure 17:**
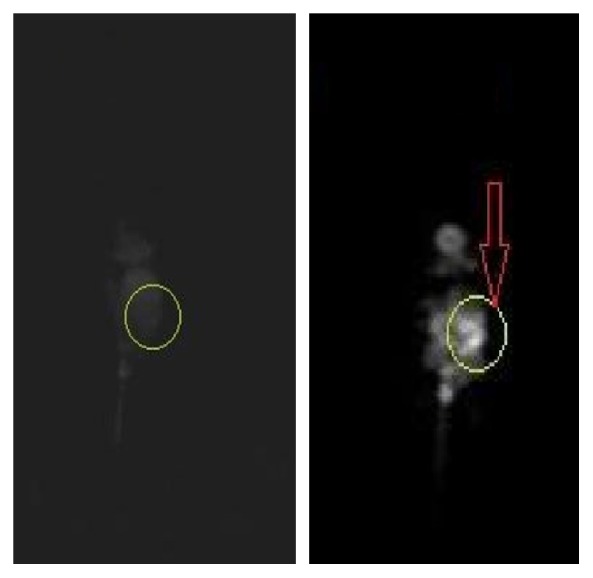
1.5 T MR image of the cancerous mice before and 60 min after injection based on T1 weighted imaging.

Figure 17 shows the MR and in vivo fluorescent images of the cancerous mice before and 60 min after Gd^3+^-dendrimer G_2_-asparagine contrast media injection. As expected the overall MRI contrast and resolution increased particularly in cancerous region. In studying, it was observed that remarkable resolution in cerebral imaging of the mice could be studied as a valuable subject in targeted cerebral imaging and brain drug delivery. The results of tumor imaging were also confirmed by in vivo fluorescent imaging 60 min after nanoconjugate injection. By performing in vivo fluorescent imaging brain resolution was not significantly detected.

### 3.17. In Vivo Efficacies

**Figure 18:**
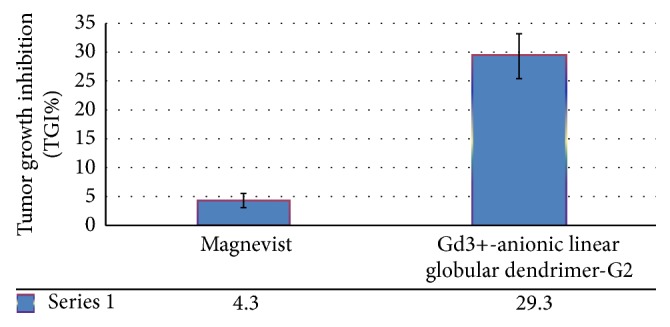
Illustration of TGI ability of nanosized Gd^3+^-anionic linear globular dendrimer-G2 compared to control groups.

This study demonstrated superior therapeutic effect of Gd^3+^-anionic linear globular dendrimer-G2 on tumor growth inhibition (TGI) compared with Magnevist (Figure 18). It is noted that as we compared our results with our current active pursuing anticancer activities of chlorambucil nanosized derivatives (unpublished data), Gd^3+^-anionic linear globular dendrimer-G2 has remarkable anticancer activity compared to chlorambucil (a well-established FDA approved anticancer drug) as well.

### 3.18. Histopathological Assessment

**Figure 19:**
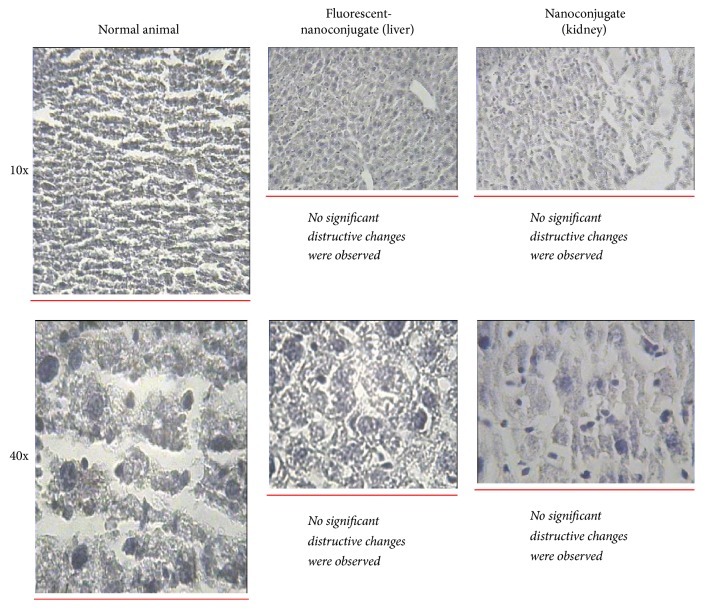
Histopathological findings of nanoconjugate receiving animal's kidney and liver 7 days after injection. No histopathological findings were observed.

No significant toxic effects regarding the use of Gd^3+^-anionic linear globular dendrimer-G2 derivatives were observed (for more details please see Figure 19).

## 4. Conclusion

In this study, for the first time, Gd^3+^-anionic linear globular dendrimer G2-asparagine, as a novel targeted molecular contrast agent for application in imaging and therapy of active tumor tissue, was introduced. Briefly such nanoconjugate was pronounced as a novel theranostic agent. In these studies, physicochemical properties, toxicity, cellular uptake, imaging, and antitumor activity studies were investigated in vitro and in vivo. By considering unique characteristics of this novel theranostic biohybrid including its inexpensive synthesis method (compared to other introduced polymers in literature such as PAMAM), biocompatibility, and improving image contrast, as well as remarkable tumor growth inhibitor, it can be concluded that it would be a suitable candidate for contrast theranostic agent for molecular imaging and therapy of cancer cells as well as brain malignancies. The results showed also an improvement in resolution in brain compared to the control group.

Current data demonstrated a very promising evidence for Gd^3+^-dendrimer G2-asparagine to be pronounced a novel theranostic radio-opaque agent which is selectively toxic on cancerous cells like HT29 as well as having no significant effects on major gadolinium contrast agent's toxic target like kidney cells.

The results of the current experiment supported different ratio of cellular uptake for Gd^3+^-dendrimer G2-asparagine in both normal and cancerous cells, respectively. Comparing the obtained cellular uptakes and cellular toxicities demonstrated that Gd^3+^-dendrimer G2-asparagine was taken up by normal cells threefold more than Magnevist as well as 17-fold more than Magnevist in cancerous cells, respectively. Additionally it is noted that cellular uptake ratio between tumor cells and normal ones illustrated threefold increase in cellular uptake ability for Gd^3+^-dendrimer G2-asparagine whereas it showed no significant decrease using Magnevist.

One of the promising mechanisms of observed cellular death for Gd^3+^-dendrimer G2-asparagine is regarding higher cellular gadolinium ion uptake because many experiments supported such hypotheses [22–28].

The cellular uptake capability of Gd^3+^-dendrimer G2-asparagine was also confirmed by flow cytometry data. According to the literature, gadolinium ions may increase cellular toxicity via activation of apoptosis pathways; these hypotheses were demonstrated by flow cytometry data. In the current experiment, we investigated further and more precisely such mechanism by employing real-time PCR and it was shown that Bax/Bcl2 genes related to apoptosis were overexpressed significantly. Our data supported more mechanistically previous findings [22–28] as well. There were two other interesting findings of the current experiment which had not yet been reported: a decrease in Magnevist cancerous cellular uptake compared to normal cells and lower toxicity of Gd^3+^-dendrimer G2-asparagine compared to Magnevist in normal cells considering more gadolinium cellular uptake for Gd^3+^-dendrimer G2-asparagine nanoconjugate.

This leads to investigating other hide mechanisms involved in cellular cell death. Furthermore, a very good brain resolution was also observed considering negative charge of our nanosized conjugate and it is very interesting.

Very promising in vitro relaxivity data in comparison to other data regarding literature was also reported for Gd^3+^-dendrimer G2-asparagine [12–17].

In conclusion, Gd^3+^-dendrimer G2-asparagine is a very promising theranostic nanosized radio-opaque agent which had shown very significant cellular uptake and tumoral toxicity as well as no significant toxic effects on human kidney cells compared to standard FDA approved drug Magnevist. Gd^3+^-dendrimer G2-asparagine can target brain and tumor as two major important areas in radiological imaging.

In brain imaging with Gd^3+^-dendrimer G2-asparagine it was previously demonstrated that asparagine has many receptors in brain like NMDA or 5-ht5A receptor [29]. Finally it is discussed that, due to lower price of generating anionic linear globular dendrimer G2 and lower toxic properties because of its negative charge and biodegradable intermediate compared to other literature dendrimers like PAMAM, producing such nanoconjugate (Gd^3+^-dendrimer G2-asparagine) as novel theranostic agent is strongly recommended based on the current presented results.

### 4.1. Future Perspective

Currently, many studies have been done on the development of nanoparticles-based on MRI contrast agent to solve the problems of traditional contrast agents with low molecular weight (e.g., rapid clearance from the blood circulation). Among the nanoparticles, dendrimers are highly considered because of their unique features such as tuning the physicochemical properties such as size, surface chemistry, solubility, biodistribution, drug release, and targeting. Dendrimers provide a high level of functional groups, multiple copies of the contrast agent, and free contact with water molecules. The use of these biodegradable dendrimers presented in this study will provide safe and effective strategy in imaging for targeting cancer cells based on metabolic demands of cancerous cells.

## Supplementary Material

SP-1: T_1_ relaxation recovery time.
SP-2: Blood clearance of Gd3+ ions of injected nano-contrast pattern. Y = -0.4814x + 86.68, R² = 0.99.

## Figures and Tables

**Table 1:** 

Asparagine	Dendrimer	Asparagine-dendrimer
0	0.534	0.604

**Table 2:** Gadolinium content measurement.

Result, mg/L	Subject test
220	Gd^3+^

**Table 3:** CHN analysis result.

Element	C	H	N
Percent	36.46%	6.99%	4.70%
Calculated number	94	216.99	10.5

**Table 4:** Signal intensity in different concentration and TR time.

TR (msec)	0.22 mg/ml	0.11 mg/ml	0.055 mg/ml	0.0275 mg/ml	0.0137 mg/ml	0.00687 mg/ml	Magnevist	Water
32	824.6	749	635	426	319.5	242	46.6	60.4
50	983.3	953.5	868.1	589.9	414.4	305.7	48.5	94
100	1225.3	1288.4	1244.9	886.4	657.6	480.1	54.9	99.6
200	1414.4	1490.4	1593.4	1257.2	1005.6	706.7	61	151.1
400	1483.5	1722.3	1854.5	1596.7	1371.2	1133.5	67.1	237.4
600	1632	1789.2	1973.8	1793	1598	1414.4	96.5	389.8
1000	1645.3	1856	2082.2	1966.8	1881.7	1725.6	111.3	537.4
2000	1676.4	1864.1	2101.1	2089	2080.1	2034	135.2	714.1
3000	1750.6	1935.8	2193.6	2110.4	20164	2164.8	174.4	1028.6
1/*T*1	0.01818	0.01360	0.009451	0.005176	0.0006312	0.001978	0.002011	0.00044
*T*1	52.91477	71.04611	115.8640	182.938449	1486.54611	494.5408622	483.3334	1595.212
Sample	1	2	3	4	5			
