# Supplementary material for: Gd3+-Asparagine-Anionic Linear Globular Dendrimer Second-Generation G2 Complexes: Novel Nanobiohybrid Theranostics
Source: Contrast Media Mol Imaging. 2017 Sep 26;2017:3625729. doi: 10.1155/2017/3625729 (PMC5635473; doi:10.1155/2017/3625729)
Supplement: Supplementary file 1 — SP-1: T1 relaxation recovery time. SP-2: Blood clearance of Gd3+ ions of injected nano-contrast pattern. Y = -0.4814x + 86.68, R² = 0.99. [file 3625729.f1.zip › Supplementary Materials.docx]

**Supporting Information (SP)**

**SP-1: T_1_relaxation recovery time**

**SP-2: Blood clearance of Gd3+ ions of injected nano-contrast pattern. Y= - 0.4814x + 86.68, R²=0.99**
